# Adenosquamous Carcinoma of the Extrahepatic Bile Duct

**DOI:** 10.31486/toj.21.0032

**Published:** 2022

**Authors:** Deepika Gupta, Sudeep Khera, Subhash Chandra Soni

**Affiliations:** ^1^ Department of Pathology and Laboratory Medicine, All India Institute of Medical Sciences, Jodhpur, India; ^2^ Department of Surgical Gastroenterology, All India Institute of Medical Sciences, Jodhpur, India

**Keywords:** *Adenosquamous carcinoma*, *common bile duct*, *extrahepatic bile duct cancer*

## Abstract

**Background:** Most malignant tumors arising from the biliary tract are adenocarcinomas. Adenosquamous carcinoma is an uncommon variant of extrahepatic bile duct cancer that is associated with more aggressive behavior than adenocarcinoma.

**Case Report:** A 58-year-old male presented with complaints of obstructive jaundice and dull aching pain in the abdomen. At his initial evaluation in another hospital, ultrasound of the abdomen and magnetic resonance cholangiopancreatography revealed common bile duct dilatation with an ill-defined lesion in the periampullary region. Endoscopic retrograde cholangiopancreatography showed infiltration of the ampulla. Biopsy specimen taken during stenting of the common bile duct suggested dysplasia. Because of recurrent jaundice, the patient was referred to our institution, and contrast-enhanced computed tomography (CT) of the abdomen showed a hypoenhancing lesion with ill-defined margins in the head of the pancreas. The patient underwent a Whipple procedure. Microscopic examination of the pancreaticoduodenectomy specimen showed an invasive adenosquamous tumor arising from the distal part of the common bile duct that infiltrated the body and head of the pancreas, involved the wall of the duodenum, and reached the duodenal mucosa. Morphologic diagnosis was supported by immunohistochemistry profile. Postoperative contrast-enhanced CT of the abdomen showed multiple enlarged mesenteric lymph nodes and multiple lesions in both lobes of the liver, suggestive of metastasis. The patient died 1 week after surgery.

**Conclusion:** Because the clinical, pathologic, and prognostic characteristics of adenosquamous carcinoma are poorly known, early diagnosis of this rare entity is warranted for patient management.

## INTRODUCTION

Adenosquamous carcinoma of the bile duct is an unusual histopathologic subtype of biliary tract cancer that is characterized by the presence of different fractions of 2 malignant infiltrative components: adenocarcinoma and squamous cell carcinoma.^1^ The amplitude of differentiation of both components fluctuates, but they tend to be moderately differentiated. Adenosquamous carcinoma can arise from the glandular epithelium of primary organs where adenocarcinomas are more prevalent, such as the breast, stomach, prostate, pancreas, colon, and rectum, but adenosquamous carcinomas have a poorer prognosis than adenocarcinomas alone.^2^ The squamous component of adenosquamous carcinoma of the bile duct is associated with more aggressive behavior than adenocarcinoma and is usually associated with invasion of local structures, tumor progression, and metastasis to distant sites.^3^ The clinical, pathologic, and prognostic features for adenosquamous carcinoma of the bile duct are not well characterized.

## CASE REPORT

A 58-year-old male with a history of diabetes mellitus type 2 for which he had been taking oral hypoglycemic medication for the prior 4 years presented to another hospital's outpatient department with yellowish discoloration of the eyes and urine that gradually progressed over a 1-month period and was associated with generalized itching and passage of clay-colored stools. He also complained of gradually progressive mild dull aching pain in his right upper abdomen that was relieved by oral analgesics. Ultrasound of the abdomen showed dilated bilobar intrahepatic biliary radicals and common bile duct dilatation up to 2 cm with abrupt distal tapering at the ampullary region. Magnetic resonance cholangiopancreatography showed an ill-defined lesion in the periampullary region (approximately 1.5 × 1.4 cm) with upstream dilatation of the common bile duct (approximately 2 cm) and bilobar intrahepatic biliary radicals. The pancreatic duct was also dilated and measured 0.6 cm. Endoscopic retrograde cholangiopancreatography (ERCP) showed infiltration of the ampulla. The common bile duct was stented, and a biopsy specimen was suggestive of dysplasia. The patient improved symptomatically following common bile duct stenting and was managed conservatively, but he was lost to follow-up during the coronavirus disease pandemic.

Three months later, the patient presented to the emergency department of another hospital with recurrent jaundice and abdominal pain. He underwent 2 sessions of ERCP and common bile duct stent exchange, one at the time of admission and another 1.5 months later. At the time of the second common bile duct stenting, contrast-enhanced computed tomography (CT) of the abdomen showed in situ common bile duct stent with pneumobilia. Side-viewing endoscopy suggested growth at the periampullary region. The patient was referred to our institution.

The patient presented to our institution 1 month later and was admitted to the gastrointestinal surgery department. At the time of admission, laboratory findings included total bilirubin 9.31 mg/dL (reference range, 0.3-1.2 mg/dL), direct bilirubin 5.63 mg/dL (reference, <0.2 mg/dL), indirect bilirubin 3.68 mg/dL (reference range, 0.1-1.0 mg/dL), serum glutamic oxaloacetic transaminase 55 IU/L (reference, <50 IU/L), serum glutamic pyruvic transaminase 42 IU/L (reference, <50 IU/L), total protein 5.06 g/dL (reference range, 6.0-8.3 g/dL), albumin 2.12 g/dL (reference range, 3.5-5.0 g/dL), globulin 2.94 g/dL (reference range, 2.5-3.3 g/dL), and alkaline phosphatase 977 IU/L (reference range, 52-171 IU/L). Kidney function tests and serum electrolytes were within normal limits. Contrast-enhanced CT of the abdomen showed a 2 × 1.3-cm hypoenhancing lesion with ill-defined margins in the head of the pancreas. The lesion was abutting the medial aspect of the second part of the duodenum, with compression of the ampulla and resultant upstream dilatation of the common bile duct and intrahepatic biliary radicals. CT also showed upper abdominal and retroperitoneal lymphadenopathy.

The patient underwent a Whipple procedure. Pancreaticoduodenectomy specimen, common hepatic artery lymph node, and 2 pericholedochal lymph nodes were submitted for histopathologic analysis. The specimen consisted of part of the distal stomach, duodenum, head of the pancreas, and common bile duct that altogether measured 30 × 6 × 4 cm. The common bile duct was dilated and measured 2 cm in diameter. A greyish-white infiltrative growth was identified in the wall of the distal common bile duct in the pancreas that extended into the body and head of the pancreas, almost reaching the ampulla and submucosa of the duodenum, and measured 3.5 × 2.8 × 1.5 cm. Grossly, the tumor was 1.2 cm from the pancreatic neck resection margin, 1.5 cm from the uncinate margin, 1.4 cm from the common bile duct cut margin, 0.9 cm from the anterior surface, 0.4 cm from the superior mesenteric vein groove, and 0.2 cm from the retroperitoneal surface of the pancreas.

**Figure 1.**
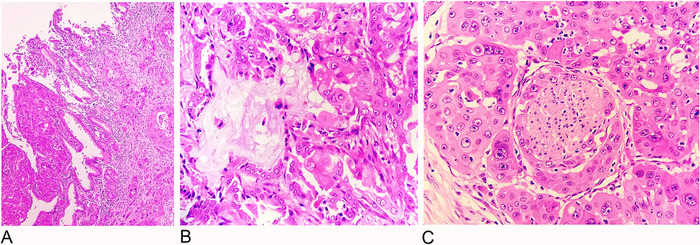
(A) Invasive and infiltrative adenosquamous tumor, arranged in glands and cords embedded in desmoplastic stroma, originated from the common bile duct mucosa (hematoxylin and eosin [H&E], magnification ×100). (B) Adenosquamous component of tumor cells shows gland formation and extracellular mucin. The glands are lined by tumor cells exhibiting moderate pleomorphism, having vesicular nuclei, conspicuous nucleoli, and a moderate amount of cytoplasm (H&E, magnification ×400). (C) Nerve shows perineural infiltration by the squamous component of the adenosquamous tumor (H&E, magnification ×400).

On microscopic examination, the mucosa of the distal part of the common bile duct was ulcerated and showed focal dysplasia from which an invasive tumor arose that infiltrated the body and head of the pancreas, involved the wall of duodenum, and reached the duodenal mucosa (Figure 1A). The tumor was arranged in glands and cords embedded in desmoplastic stroma. These glands were lined by tumor cells showing moderate to marked pleomorphism and having vesicular nuclei, conspicuous nucleoli, and a moderate amount of cytoplasm with extensive squamous differentiation (Figure 1B). Bizarre cells and multinucleated tumor giant cells were seen, along with occasional mitotic figures. Some of the glands showed mucinous secretion. Focal necrosis was also noted. Lymphovascular invasion and extensive perineural invasion were present (Figure 1C). All resection margins and surfaces were free from tumor invasion.

Of the 14 lymph nodes retrieved from the pancreaticoduodenectomy specimen, 7 showed tumor deposits and 1 showed extranodal extension. The common hepatic artery lymph node did not show any evidence of tumor deposit. One of the 2 pericholedochal lymph nodes showed tumor deposits.

**Figure 2.**
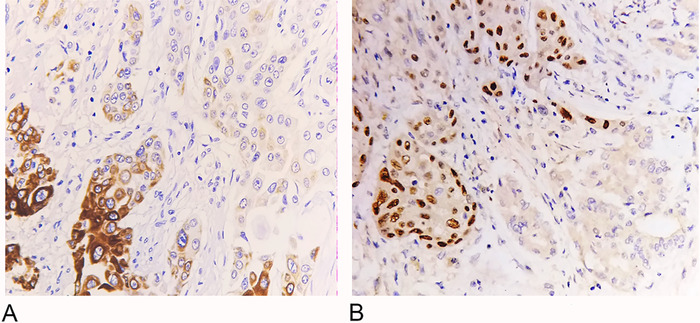
(A) Adenocarcinoma component shows immunoreactivity for CK19 (immunohistochemistry [IHC], magnification ×400). (B) Squamous cell carcinoma component shows immunoreactivity for P40 (IHC, magnification ×400).

On immunohistochemistry, the adenocarcinoma component was positive for CK7 and CK19 (Figure 2A), and the squamous component was positive for P40 (Figure 2B). Based on the morphologic and immunohistochemical findings, extrahepatic cholangiocarcinoma, adenosquamous type, moderately differentiated (pT3N2) was diagnosed.

Postoperative contrast-enhanced CT of the abdomen showed multiple enlarged mesenteric lymph nodes and multiple lesions in both lobes of the liver, suggestive of metastasis. Chemotherapy was planned, but the patient died 1 week after surgery.

## DISCUSSION

Primary carcinoma of the bile duct is an uncommon tumor, with an autopsy incidence of 0.3%; more than 95% are ductal adenocarcinomas principally occurring in the extrahepatic biliary tree.^4^

Adenosquamous carcinoma of the biliary tract accounts for 0.6% to 2% of all biliary tract malignancies,^1,2^ and adenosquamous carcinoma of the extrahepatic bile duct has been documented to represent approximately 2.2% to 4.7% of extrahepatic bile duct carcinomas.^5,6^ According to the 2019 World Health Organization (WHO) classification of tumors of the digestive system, the most common carcinomas of the extrahepatic bile duct are pancreatobiliary-type adenocarcinomas, and the rare types include squamous cell carcinoma, adenosquamous carcinoma, sarcomatoid carcinoma, and undifferentiated carcinoma.^1^ Adenosquamous carcinoma is a specific histopathologic variant, regardless of its glandular origin. WHO has defined adenosquamous carcinoma of the gallbladder as one in which the squamous elements constitute at least 25% of the tumor; however, no minimum percentage of the squamous cell carcinoma component is required for the diagnosis of adenosquamous carcinoma of the extrahepatic bile duct.^1^ The clinical, pathologic, and prognostic characteristics of extrahepatic bile duct adenosquamous carcinomas remain unclear because of their rarity and the scarcity of reported cases.

Although the tumor etiology is uncertain, theories have been proposed for the histogenesis of adenosquamous carcinoma. One theory is malignant transformation of the bile duct epithelium from an initially benign squamous metaplasia of the epithelium subsequent to a chronic inflammatory process because of gallstones, infection, or a choledochal cyst.^7^ Another and more reliable theory is the squamous metaplasia or transformation of the persistent adenocarcinoma itself.^8^ Yet another potential mechanism is the capability of pluripotent stem cells to induce transformation of both adenocarcinoma and squamous cell carcinoma or the collision of both types of tumor.^9^

Grossly and radiologically, adenosquamous carcinoma of the extrahepatic bile duct does not present as a bulky mass, in contrast to adenosquamous carcinomas of the gallbladder and liver which frequently manifest as bulky masses and at an advanced stage. Because the extrahepatic bile duct is anatomically localized at a very narrow space, extrahepatic bile duct carcinomas present early with symptoms of obstructive jaundice.^10^

Most studies of adenosquamous carcinoma of the bile duct are either case reports or small case series (Table)^2,3,10-15^ because of its low prevalence. Qin et al studied 106 patients with adenosquamous carcinoma of the bile duct during a span of 40 years.^2^ Fifty-eight patients had lesions of the extrahepatic bile duct, and 34 patients had lesions located at the ampulla of Vater. Qin et al found the 1-year, 2-year, and 5-year overall survival (OS) for patients with adenosquamous carcinoma of the bile duct was 30.1%, 11.3%, and 3.7%, respectively, and the median OS after cancer-directed surgery was 14 months for ampulla of Vater cases, 6 months for extrahepatic bile duct cases, and 6 months for intrahepatic bile duct cases. Qin et al concluded that cancer-directed surgery provides an additional 10 months of OS for patients with adenosquamous carcinoma of the bile duct.^2^

**Table.** Reported Cases of Adenosquamous Carcinoma of the Extrahepatic Bile Duct

Study	Number of Cases	Patient Age, Years	Patient Sex
Lantsberg et al, 1986^13^	1	83	F
Okabayashi et al, 2005^12^	1	55	M
Lim et al, 2007^14^	1	83	M
Hong et al, 2008^11^	12	48-78 (mean, 60)	F: 4
			M: 8
Kim et al, 2009^10^	6	49-71 (mean, 64)	F: 1
			M: 5
Aoki et al, 2012^15^	1	83	F
Hoshimoto et al, 2017^3^	4	68-74 (mean, 72)	F: 2
			M: 2
Qin et al, 2018^2^^,^^a^	58	Not reported	Not reported
Present case, 2022	1	58	M

^a^The study includes 106 cases of adenosquamous carcinoma of the bile duct, of which 58 patients had tumors of the extrahepatic bile duct, 34 of the ampulla of Vater, 6 of the intrahepatic bile duct, and 8 of the bile duct, not otherwise specified. The mean age at diagnosis was 68.1 ± 13.5 years for all patients with adenosquamous carcinoma of the bile duct, but the authors do not provide the ages of patients with adenosquamous carcinoma of the extrahepatic bile duct.

F, female; M, male.

Literature detailing the clinical outcomes of patients with adenosquamous carcinoma of the extrahepatic bile duct is scarce compared to that of adenosquamous carcinoma of the gallbladder. A study of 12 cases of adenosquamous carcinoma of the extrahepatic bile duct demonstrated that patients tend to have frequent invasion of the duodenum, as well as deeper invasion, more advanced disease stage, and poorer survival rates than patients with adenocarcinoma, with 1-, 3-, and 5-year survival rates of 46%, 18%, and 9%, respectively.^11^

The aggressive nature of adenosquamous carcinoma has been attributed to the proliferative capacity of the squamous component that causes the carcinoma to exhibit a high degree of malignancy.^9,12^ In a study by Hoshimoto et al of 172 patients with biliary tract cancer (40 patients with gallbladder carcinomas, 105 with extrahepatic bile duct carcinomas, and 27 with ampulla of Vater carcinomas), only 9 cases (5.2%) were pathologically diagnosed as adenosquamous carcinoma: 4 cases in the gallbladder, 4 cases in the extrahepatic bile duct, and 1 case in the ampulla of Vater.^3^ Hoshimoto et al concluded that the squamous cell carcinoma component of adenosquamous carcinoma in the biliary tract displayed a relatively higher proliferative ability, which might be associated with local invasiveness. They found a preponderance of the squamous cell carcinoma component in the advancing region of the tumor—angiolymphatic and perineural invasion in most of the cases—but the proportion of the squamous cell carcinoma component was decreased in the metastatic sites in more than half of the cases.^3^ In our case, the lesion originated from the distal common bile duct and was locally infiltrative, with pancreatic, serosal, perineural, and lymphovascular invasion. The perineural infiltration was principally caused by the squamous component rather than the glandular component of the tumor.

Patients with adenosquamous carcinoma have a better prognosis than patients with squamous cell carcinoma but a less favorable prognosis than patients with adenocarcinoma. Although the biologic behaviors of adenosquamous carcinoma and adenocarcinoma of the bile duct are different, treatment of patients with adenosquamous carcinoma is similar to that for patients with adenocarcinoma.

Because adenosquamous carcinomas are rare tumors, the results of different therapeutic strategies are not well understood, and no therapeutic intervention standards have been provided. Qin et al found a longer OS for patients who received surgery,^2^ but the benefit of radiotherapy and chemotherapy as adjuvant treatments for patients with operated biliary tract tumors remains under investigation. Hong et al reported effective results of treatment with trastuzumab, chemotherapy, and radiotherapy of adenosquamous carcinoma of the biliary tree with HER2 overexpression.^16^

## CONCLUSION

Adenosquamous carcinoma of the extrahepatic bile duct is a rare histologic variant with a poor prognosis. The literature indicates that overall survival is decreased when the percentage of the squamous cell carcinoma component increases; therefore, identification of the squamous cell component has important prognostic implications as the patient can be started early on other treatment modalities after the surgery.

## Notes

*This article meets the Accreditation Council for Graduate Medical Education and the American Board of Medical Specialties Maintenance of Certification competencies for Patient Care and Medical Knowledge.*
